# Optimization of Subcritical Water Extraction (SWE) of Lipid and Eicosapentaenoic Acid (EPA) from* Nannochloropsis gaditana*

**DOI:** 10.1155/2018/8273581

**Published:** 2018-12-27

**Authors:** Bernard Chon Han Ho, Siti Mazlina Mustapa Kamal, Michael K. Danquah, Razif Harun

**Affiliations:** ^1^Department of Chemical and Environmental Engineering, Faculty of Engineering, Universiti Putra Malaysia, 43400 Serdang, Malaysia; ^2^Department of Process and Food Engineering, Faculty of Engineering, Universiti Putra Malaysia, 43400 Serdang, Malaysia; ^3^Department of Chemical and Petroleum Engineering, Curtin University, Sarawak, Malaysia

## Abstract

Microalgae are a promising source of omega-3. The purpose of this study was to extract lipid with a relatively high content of eicosapentaenoic acid (EPA) from* Nannochloropsis gaditana *using subcritical water extraction (SWE). The effects of different temperatures (156.1-273.9°C), extraction times (6.6-23.4 minutes), and biomass loadings (33-117 g algae/L) on the extraction yield were studied. From the optimization study using central composite design (CCD), quadratic models generated for lipid yield and EPA composition were considered to be significant models (*p* < 0.05). The predictive equations were also formed for lipid yield and EPA composition. The predicted optimum lipid yield and EPA composition at 236.54°C, 13.95 minutes, and 60.50 g algae/L were 18.278 wt% of total biomass and 14.036 wt% of total fatty acid methyl ester (FAME), respectively.

## 1. Introduction

Optimum health and quality of life have been key priorities for every citizen in the world. Omega-3 plays a significant role in the body's inflammatory pathways and cell health, especially for cancer prevention and therapy [1–3]. Omega-3 fatty acids such as docosahexaenoic acid (DHA) and eicosapentaenoic acid (EPA) are categorized under long-chain polyunsaturated fatty acids (PUFAs). DHA and EPA provide many health benefits that include the prevention of cardiovascular diseases (CVD) [4], treatment of depression patients [5], and improvement of many diseases such as attention deficit hyperactivity disorder (ADHD) [6], mild cognitive impairment [7], and Alzheimer's [8].

With current research and development interest, microalgae could step up as one of the potential sources for future generation of omega-3. Currently, the utilization of microalgae for various bioproducts and sustainable energy production has been vastly reported [9, 10]. Characteristics that made microalgae interesting for commercial applications are their capacity to produce high biomass yield per unit of light and area and high biooil content while minimizing the harvesting cycle time and the usage of arable land [11]. However, some researchers had overlooked the bioavailability of algal oil especially in terms of omega-3. Many of the algae species have been reported to be high in omega-3 content, especially in terms of EPA and DHA [12–14]. Based on the literature,* Nannochloropsis gaditana* has been identified to have high total lipid and EPA content of up to 24 wt% of total biomass and 32 wt% of total fatty acid methyl ester (FAME), respectively [12]. Microalgae are also classified as kosher and a vegetarian source of DHA and EPA. With the given benefits, microalgae could be incorporated into fortified food and consumed as daily supplements. Some companies such as OmegaTech (USA) have started to include algae DHA into adult dietary supplements and animal feed [15].

The extraction of lipid containing omega-3 from microalgae remains a challenge. Most conventional extraction techniques involve complicated procedures and long processing time with the utilization of hazardous solvents [16]. These hinder the application of the extracted products to be fully utilized for human consumption. Therefore, there is a need for a green, fast, and robust approach to extract lipids from microalgae. Subcritical water extraction (SWE) is a green technique to extract bioactive compounds and lipid from biomass. It uses water as extraction solvent hence eliminating the use of harmful solvents. SWE is performed using high temperature water (above boiling point) and pressure at the subcritical region to keep the water in the liquid state [17]. It is very effective in hydrolyzing the cell wall of biomass and solubilizing the bioactives thus enhancing the extraction process of bioactive components. This technology provides lower production cost, milder operating conditions, and a shorter production period compared to other conventional methods such as chemical and biological [18]. Although this method provides a positive outlook, the utilization of the SWE method in optimizing the extraction of omega-3 from microalgae is scarcely reported. This is due to the fact that some of the researchers are more interested in the conversion of microalgal lipid into biofuel rather than converting the lipids for omega-3 production. For instance, Reddy et al. [19] and Sitthithanaboon et al. [20] reported that the extracted lipid from* Nannochloropsis* contained high EPA but they did not conduct optimization on the fatty acid compound as their studies only focused on biodiesel production. Meanwhile, supercritical fluid extraction also has a great potential to be used for the extraction of omega-3 and lipid from microalgae [21, 22]. However, the mechanism of supercritical fluid extraction might increase the difficulty for this system to be scaled up and induce high maintenance cost [23].

Therefore, this article investigates the effect of different extraction parameters of SWE including temperature, extraction time, and biomass loading on the lipid yield and EPA composition extracted from microalga* Nannochloropsis gaditana*. An optimization study through central composite design (CCD) was done to predict the optimum yield for lipid extract and EPA composition. With CCD used in this work, the problem of single-factor study that does not reflect the interaction of all factors could be overcome. Meanwhile, the optimum yield of the desired products could be obtained through the response surface methodology (RSM) approach while reducing the number of experiments to be conducted. Hence, the experimental cost and time consumption could be significantly reduced. Many researchers had incorporated this optimization method study into their researches and had successfully shown the interactions of all the studied parameters [24–26].

## 2. Materials and Methods

### 2.1. Strain and Characterization

*Nannochloropsis gaditana* was purchased and shipped from Longevity Superfoods (Utah, USA) in powdered form. The biomass was freeze-dried and stored in resealable bags until further usage. The total carbon, hydrogen, nitrogen, and sulfur (CHNS) analysis was done using the CHNS 628 elemental analyzer (LECO, USA). The calibration for CHN and S was done using 0.1 g EDTA standard (LECO, USA) and 0.1 g coal standard (LECO, USA), respectively. Then, 0.2 g of the biomass was transferred to the CHNS analyzer for ultimate analysis and the analysis was performed in duplicate except for sulfur analysis. The lipid content of the biomass was identified using the conventional setup of the Soxhlet extraction method. N-hexane purchased from Alpha Lab Solution Resources, Malaysia, was used as the solvent and the process was carried out for 8 hours. Protein content was determined using the Kjeldahl method whereas the carbohydrate content was taken as the sum of residuals after all the other compounds were determined. The moisture content of the biomass was determined by drying the biomass in the oven at 105°C until the mass was constant and incinerated at 550°C to determine the ash content. The presence of moisture content might be due to the surrounding moisture being absorbed by the sample. All the procedures were done in duplicate and the average readings were reported. The energy content was calculated according to the method reported by Bhattacharjee et al. [27].

### 2.2. Subcritical Water Extraction

**Figure 1:**
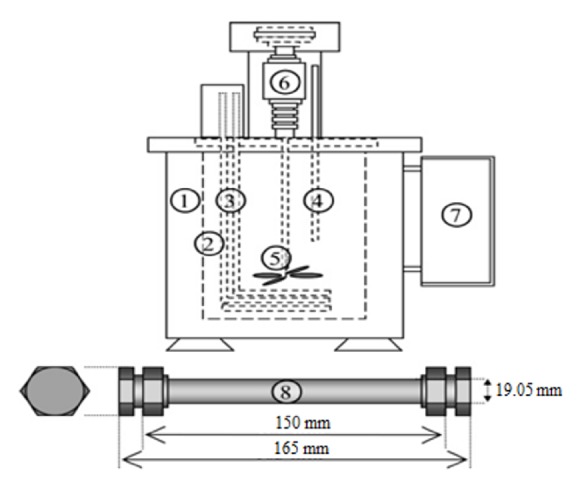
Schematic diagram of subcritical water extraction setup (1. isolation chamber; 2. inner salt bath; 3. heater (4000 W); 4. temperature sensor; 5. mixer; 6. stirring motor; 7. operation panel; 8. stainless steel reactor).

The subcritical water setup is shown in Figure 1. The main components of the system consist of a salt bath where the salt used was a mixture of potassium nitrate (R&M Chemicals, Malaysia) and sodium nitrite (R&M Chemicals, Malaysia) in a ratio of 1:1, a temperature controller unit, and a 32 mL stainless steel reactor (Swagelok 316 SS; 3/4 in. outer diameter × 0.049 in. wall).

The stainless steel reactor was filled with the desired amount of freeze-dried biomass and distilled water (33-117 g algae/L) and mixed using a vortex mixer. Then, the reactor was purged with pure argon for approximately 5 seconds to remove any air impurities entrapped inside the reactor and tightened immediately. The reactor was then placed horizontally into the salt bath with the desired temperature (156.1-273.9°C) and extraction time (6.6-23.4 minutes). The temperature was controlled using a thermostatic salt bath with an accuracy of ±0.1°C. The cooling of the reactor was performed in a room temperature water bath before opening the reactor. After each extraction, the extract was poured into a centrifuge tube and the reactor was washed with 10 ml of n-hexane to recover crude lipid that was left inside the reactors. The centrifuge tube containing the extracts and n-hexane was centrifuged at 2500 x* g* for 10 minutes using a compact tabletop centrifuge (KUBOTA 2420). A glass Pasteur pipette was used to recover the top layer of the crude lipid. The recovery procedure using n-hexane was repeated for 3-4 times to complete the separation of crude lipid from the aqueous phase. The n-hexane and crude lipid mixture was then transferred to a rotary evaporator for final separation of crude lipid from n-hexane. The weight of the obtained crude lipid was measured and the lipid was stored in a freezer (-20°C) for further analysis. The residual extracted algae were washed with distilled water and dried at 60°C. The microstructures of the dried untreated and extracted microalgae were analyzed with FEI Nova NanoSEM 230 Field Emission-Scanning Electron Microscopy (FE-SEM) operated at 50 kV. The samples were sputter-coated with gold prior to SEM analysis.

### 2.3. Gas Chromatography Analysis

1 mL of n-hexane was added to the crude lipid extract. Another 1 mL sodium methoxide solution (1 mg/ml) (Sigma-Aldrich (M) Sdn. Bhd., Malaysia) was added to the mixture and the solution was stirred using a vortex stirrer for 10 seconds. The solution was allowed to settle for 10 minutes for methanolysis reaction to take place and the top FAME layer was collected for GC analysis [28].

The FAME was analyzed using a Hewlett-Packard 5890 Series II Plus gas chromatograph, equipped with an automatic liquid sampler and flame ionization detector (FID). The column used was DB 225 (30 m × 0.25 mm × 0.25 *µ*m) purchased from Agilent, USA, with a stationary phase of (50%-cyanopropylphenyl)-dimethylpolysiloxane. The carrier gas was hydrogen and the inlet pressure was at 20 psi. The injection was in splitless mode with an injection volume of 1.0 *µ*L. The initial oven temperature was at 35°C and was held for 0.5 min. Then, with a heating rate of 25°C/min, the temperature reached 195°C after 6.4 min. Then, the rate was changed to 3.0°C/min until the oven temperature reached 205°C and to 8.0°C/min reaching the final temperature of 230°C. Finally, the temperature at 230°C was held for 6.70 min. The total run time was 20.06 min. The detector temperature was set to 240°C and the makeup gas used was nitrogen gas. The retention times of the samples' peaks were compared to the Supelco® 37 Component FAME Mix standard (Sigma-Aldrich (M) Sdn. Bhd., Malaysia) and quantified as the percentage area of each component of FAME.

### 2.4. Experimental Design

The interactions of three factors (temperature, extraction time, and biomass loading) on the lipid yield and EPA composition were studied using RSM. The experimental cost and time would be reduced with the implementation of a mathematical model, aiming to reduce the number of experiments. The mathematical model used in this study was CCD with three variables. To make the design rotatable, the star points were included into the design as “alpha” and set at 1.68 in coded units which were the axial distance from the center point. The high and low settings for the design were chosen according to the work done by Reddy et al. [19].  Table 1 shows the coded and selected values for all the studied factors. The total number of experimental runs was 17 inclusive of 3 replications at the center point as shown in Table 2. The experimental runs were conducted in random order as generated by Design-Expert® (Version 6) to minimize or balance the effects of external factors that can impact the results. The model was fitted to a common second-order polynomial model:

(1) $$\begin{array}{c} Y=\beta_{0}+\overset{3}{\underset{i=1}{\sum }}\beta_{i}x_{i}+\overset{3}{\underset{i=1}{\sum }}\beta_{ii}{x_{i}}^{2}+\overset{2}{\underset{i=1}{\sum }}\overset{3}{\underset{j=i-1}{\sum }}\beta_{ij}x_{i}x_{j}, \end{array}$$

where* x*_1_,* x*_2_, and* x*_3_ are the levels of the three factors; *β*_0_ is the constant coefficient; *β*_i_ is the linear coefficient; *β*_ii_ is the quadratic coefficient; *β*_ij_ is the cross product coefficient. The 3-dimensional surface plots were developed using Design-Expert® (Version 6). The validity of the predicted response was checked using complete analysis of variance (ANOVA) of the quadratic models developed. With the optimization results, the optimized lipid yield and EPA composition will be obtained.

## 3. Results and Discussion

### 3.1. Proximate Analysis and Ultimate Composition

Microalga* Nannochloropsis gaditana* was used as the biomass for omega-3 production. This species has relatively high EPA content and moderate lipid content compared to other high omega-3 microalgae [12]. The elemental and biochemical composition of the biomass is shown in Table 3.

Based on Table 3, the high crude protein resulted in high nitrogen content of 7.54 wt%. The ratio of H:C multiplied by a factor of 10 against the ratio O:C of this sample was 1.45 against 0.70 which falls in the biomass region of the Van Krevelen diagram [29]. The elemental composition of the biomass was also consistent with the other* Nannochloropsis gaditana* reported in the literature [30]. The biomass has relatively lower lipid content of 10.2 wt% as compared to the protein concentration of 47.2 wt%. Even so, some researchers had proven that SWE was able to provide high recovery on the crude lipid extract on low lipid microalgae [12, 31].

### 3.2. Soxhlet and SWE Yield

GC-FID analysis was done using the extracted lipid from the Soxhlet extraction method. Note that the Soxhlet was used as a control method of lipid extraction and further compared with the SWE method. Based on the analysis, EPA composition extracted by Soxhlet was 28.103 wt% of the total FAME. Rios et al. [12] reported that there is a possibility of Soxhlet to extract lipid with a higher composition of EPA from* Nannochloropsis gaditana* as compared to other extraction methods. However, the low amount of lipid extracted in this study showed that the Soxhlet extraction did not completely extract the lipid content from the microalgae [32].

Meanwhile, for SWE, the lipid yield and EPA composition were 17.860 wt% of total biomass and 14.135 wt% of total FAME, respectively. These were achieved when the biomass was extracted at 215°C, 15 mins, and 75 g algae/L (Run 15) as shown in Table 4. The higher lipid content extracted from SWE was due to the altering of the polarity of water in subcritical water condition, allowing both polar and nonpolar lipid to be extracted from the microalgae [33]. Although SWE might improve the extraction of lipid from microalgae, there was a change in the composition FAME. The composition of a higher PUFA composition was shifted to a higher saturated fatty acid (SFA) composition after SWE. This might be due to the instability of long-chain FAME and PUFA at higher temperature [34]. Hence, there is a need for the optimization run to be conducted.

### 3.3. Analysis of Model

Based on the ANOVA, it was found that the response for lipid yield (*Y*_1_) did not require any transformation but the response for EPA composition (*Y*_2_) was recommended to undergo inverse square root transformation as suggested by the model. The standard deviation (*σ*) of *Y*_2_ was reduced from 2.39 to 0.03 after inverse square root transformation. According to Ajibade et al. [35], the condition of successful inverse square root transformation was *σ* ≤ 0.14. The condition is well matched with the findings of this study. The detailed ANOVA for both *Y*_1_ and *Y*_2_ are shown in Tables 5 and 6, respectively. The quadratic model was used because the model was considered significant (*p* < 0.05) for both responses.

Meanwhile, the significance of the terms in the quadratic model was determined based on the* p* value (<0.05) coded as A, B, and C for temperature, extraction time, and biomass loading, respectively. For lipid yield model, the significant terms were A, C, A^2^, B^2^, C^2^, and the interaction of B and C. The term B was added after the backward elimination regression even though it is a nonsignificant term due to the hierarchy of the term [36]. For EPA composition, the terms A, C, and A^2^ were significant. The predictive equations for two of the response variables are given below:

(2) Y1=−81.93777+0.74454A+1.70040B+0.056868C−1.58371×10−3A2−0.084720B2−1.97039×10−3C2+0.010960BC

(3) Y2−0.5=3.09492−0.023396A−7.68857×10−4C+4.91963×10−5A2.

The deviation or variation about the mean and predictive capability of the model were also tested and shown in the ANOVA table as shown in Tables 5 and 6. The R^2^ values (> 0.90) for both models showed good fit of the data to the fitted regression line. The adjusted-R^2^ value of about 0.88 for both models also showed the models were not too complex for the sample size and the number of variables was sufficient. This could reduce the biasness of the model and increase the precision of the estimated coefficients. Meanwhile, the predicted-R^2^ showed an acceptable predictive strength to the model as the predicted R^2^ were still in reasonable agreement with the adjusted R^2^ of the models. The experimental and predicted values for the responses were shown in Table 2 and the predictions were reasonably close to the experimental values. Also, both of the models showed adequate signals and nonsignificant lack of fit where the adequate precision ratio was larger than 4 and lack of fit was more than 0.05. Hence, the models were suitable to be used to navigate the design space and the model was considered to fit.

### 3.4. Interactions and Influences of Process Variables

**Figure 2:**
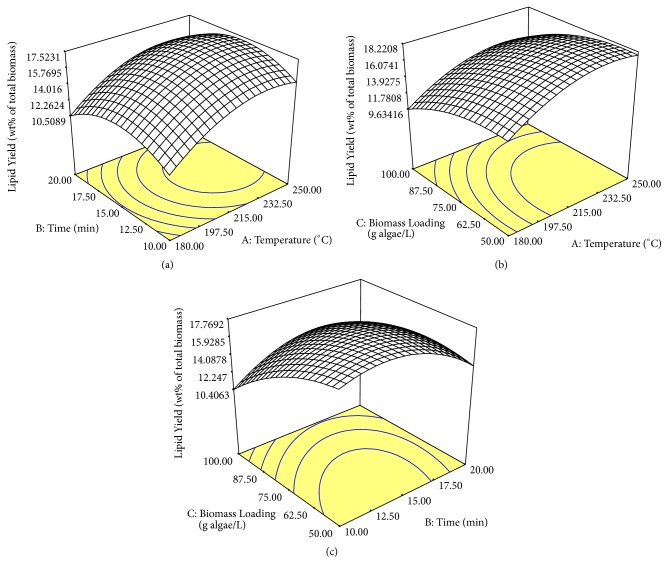
The interaction of all the factors of SWE on lipid yield using RSM with set factors of (a) biomass loading = 75 g algae/L, (b) time = 15 min, and (c) temperature = 215°C.

**Figure 3:**
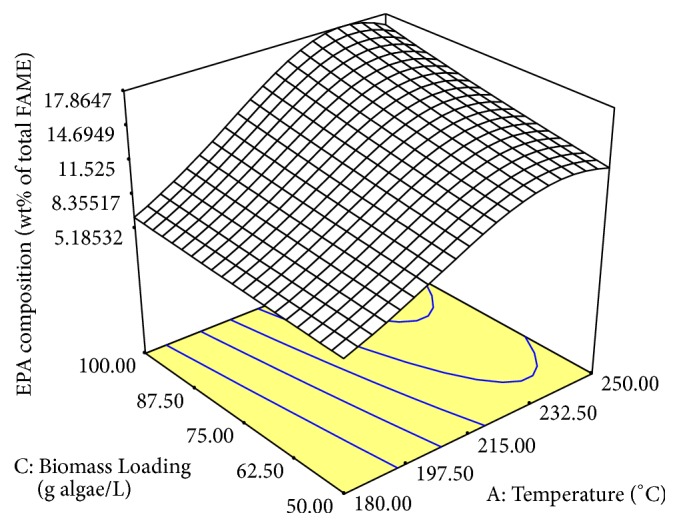
The interaction of significant factors of SWE on EPA composition using RSM.

**Figure 4:**
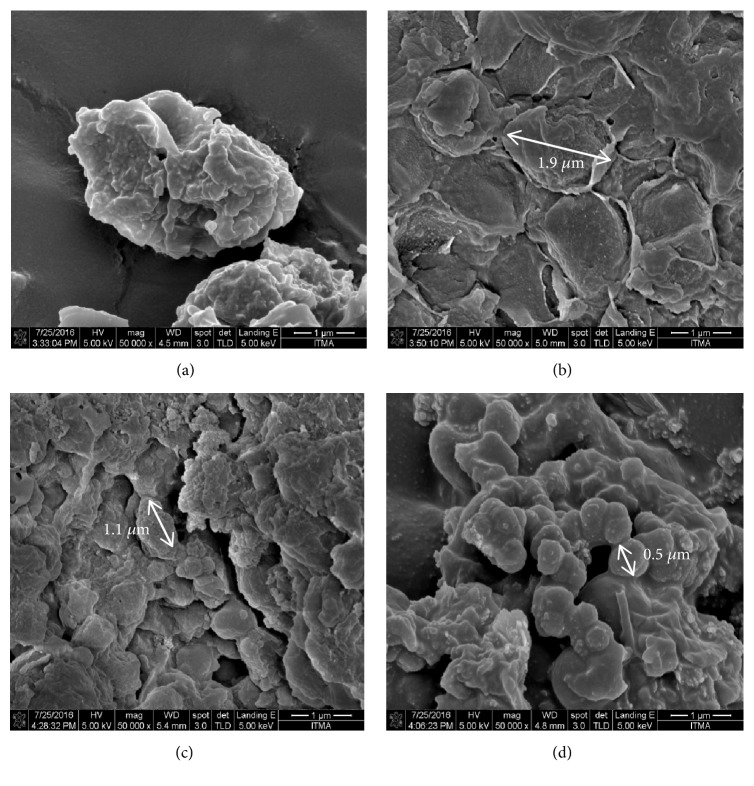
SEM images of (a) untreated microalgae and extracted microalgae at 15 minutes and 75 g algae/L with different temperatures of (b) 156.1°C, (c) 215.0°C, and (d) 273.9°C.

The 3-dimension (3D) surface plot in Figures 2 and 3 showed the interaction of the statistically significant factors. In Figures 2 and 3, the changes in temperature clearly showed a significant change to the lipid yield and EPA composition, respectively. The lipid yield and EPA composition increased with the increasing temperature until the optimum point was reached. Then, the lipid yield and EPA composition decreased with the increasing temperature towards 250°C. SWE was effective in extracting lipid and EPA at high temperature due to the increase in the solubility of water in nonpolar species [17]. However, according to Reddy et al. [19], hydrolysis is the predominant reaction in the temperature range above 240°C. This is due to the increase in reactivity of water as the condition moves closer to the critical point [37]. Degradation of lipid components might occur in the higher temperature region thus reducing the lipid yield and EPA composition [38]. From Figure 4, the microstructure of algae at a low temperature of 156.1°C showed that the cell wall of the microalgae began to break. Ma et al. [39] studied that the degree of rupturing of microalgae cell influenced the extraction yield of the lipid in the cell. At a temperature of 215°C, the degree of rupturing increased resulting in an increase of lipid yield. However, at a high temperature of 273.9°C, the size of the microalgae became smaller at approximately 0.5 *µ*m. Complete rupturing happened at high temperature resulting in the formation of clusters of cells where individual cells could not be distinguished. Hence, there might be possibilities of degradation of extracted products as the high temperature was high enough to cause the deformity in the morphology of the cells.

Extraction time in SWE played a significant role in extracting lipid but not in the extraction of EPA. As shown in Figures 2(a) and 2(c), the extraction time significantly changed the lipid yield where there was a significant optimum time for the highest lipid yield. Meanwhile, extraction time within the studied range was not statistically significant in affecting the EPA composition. However, the increase in exposure of the subcritical water condition in the reactor allows secondary or tertiary reactions to happen [38]. This will reduce the quality and the amount of the extracted lipid. The deterioration of the quality of lipid could be observed with the interaction between the extraction time and temperature.

Another variable that affects the lipid extraction of* Nannochloropsis gaditana* was biomass loading. Biomass loading is an important factor to study as dewatering technology currently contributes to high energy consumption hence leaning toward high costs for the overall process [40]. Therefore, low biomass loading or high water content processing is considered to be preferable at current industrial practices. From Figures 2(b) and 2(c), the reduction in biomass loading or the increase in water content as compared to algae ratio increased the lipid yield. This clearly showed that the increase in the amount of water was sufficient to hydrolyze the algal cell wall and solubilize the lipid content in the biomass. Meanwhile, the increase in biomass loading increased the extraction of EPA as shown in Figure 3. The low EPA composition in low biomass loading might be due to the increased degradation of EPA as compared to the other PUFAs. Hence, the percentage of EPA in total FAME decreased as compared to other PUFAs. Long-chain omega-3 fatty acids are more susceptible to oxidation when compared to other shorter-chain fatty acids [41].

### 3.5. Optimization of the Extraction Model

The optimization of the extraction model for both lipid and EPA yields was obtained from Design-Expert®. The conditions were preset to the desired product yield. In Table 7, the maximum lipid yield of 18.408 wt% could be obtained at 235.04°C, 13.38 minutes, and 51.71 g algae/L of water. However, the maximum lipid yield compromised the content of EPA in the lipid and the EPA was predicted to yield 13.325 wt% of total FAME. Meanwhile, the maximum EPA yield could be as high as 17.887 wt% of total FAME as predicted by the process condition as shown in Table 7. Hence, both of the process responses (lipid yield and EPA composition) were taken into consideration when optimizing the SWE process. With both desired conditions of maximum lipid yield and EPA composition, the optimum process conditions were predicted at 236.54°C, 13.95 minutes, and 60.50 g algae/L, predicting the optimum lipid and EPA content of 18.278 wt% of biomass and 14.036 wt% of total FAME, respectively. An additional independent experiment was done at optimum condition, yielding total lipid and EPA content of 13.405 wt% of total biomass and 15.040 wt% of total FAME, respectively. The experimental error which is less than the acceptable range of 5% implied that the predicted and experimental results are in good agreement.

## 4. Conclusions

In this study, the RSM with CCD was successfully used in developing a model for SWE of* Nannochloropsis gaditana. *The interactions of all process variables (temperature, extraction time, and biomass loading) were clearly shown in the model analysis. The high lipid yield obtained from SWE proved that the extraction efficiency was comparable to conventional solvent extraction. Meanwhile, the study to improve the selectivity of EPA needs to be done more extensively. The substitution of the long and harmful process of solvent extraction with green extraction such as SWE is very important in the future. With a good omega-3 extraction prospect, the SWE technology should be further enhanced and studied to improve the quality of lipid extracts.

## Figures and Tables

**Table 1:** Coded values and process conditions for CCD of SWE of *Nannochloropsis gaditana*.

Coded Values	-*α*	-1	0	+1	+*α*
Temperature, A (°C)	156.1	180.0	215.0	250.0	273.9
Extraction time, B (min)	6.59	10.00	15.00	20.00	23.41
Biomass loading, C (g/L of water)	32.96	50.00	75.00	100.00	117.04

**Table 2:** Experimental design matrix and responses of SWE of *Nannochloropsis gaditana*.

Standard order	Run order	Temperature, A (°C)		Extraction time, B (min)		Biomass loading, C (g algae/L)		Lipid yield (wt% of total biomass)		EPA composition (wt% of total FAME)	
		Process	Coded	Process	Coded	Process	Coded	Experimental	Predicted	Experimental	Predicted
6	1	250	+1	10	-1	100	+1	9.265	10.690	14.425	16.825
8	2	250	+1	20	+1	100	+1	13.880	13.238	13.939	16.825
11	3	215	0	6.59	-*α*	75	0	12.053	10.995	15.784	12.454
4	4	250	+1	20	+1	50	-1	15.560	14.213	13.615	12.554
2	5	250	+1	10	-1	50	-1	16.420	17.145	12.483	12.554
7	6	180	-1	20	+1	100	+1	9.880	8.790	7.4837	6.228
3	7	180	-1	20	+1	50	-1	8.870	9.765	4.065	5.185
12	8	215	0	23.41	+*α*	75	0	9.513	10.733	8.855	12.646
5	9	180	-1	10	-1	100	+1	7.535	6.242	5.868	6.228
14	10	215	0	15	0	117.04	+*α*	9.407	10.313	14.889	15.657
17	11	215	0	15	0	75	0	15.667	16.887	19.365	12.646
1	12	180	-1	10	-1	50	-1	12.010	12.697	4.783	5.185
10	13	273.9	+*α*	15	0	75	0	15.313	15.136	10.389	9.775
13	14	215	0	15	0	32.96	-*α*	17.175	16.528	11.287	10.173
15	15	215	0	15	0	75	0	17.860	16.887	14.135	12.646
9	16	156.1	-*α*	15	0	75	0	7.260	7.650	3.155	2.933
16	17	215	0	15	0	75	0	17.173	16.887	13.155	12.646

**Table 3:** Elemental composition, energy value, and proximate analysis of *Nannochloropsis gaditana*.

C	H	N	S	O*∗*	Energy (MJ/kg)	Crude lipid	Moist. content	Carb.*∗*	Ash	Crude protein
(wt%)						(wt%)				
48.12	6.95	7.54	0.63	36.77	16.592	10.2	2.9	28.9	10.8	47.2

*∗* : by difference

**Table 4:** Fatty acid methyl ester composition from Soxhlet (8 hours using n-hexane) and SWE extraction (Run 15 at 215°C, 15 mins, and 75 g algae/L).

**Fatty Acid Methyl Ester (FAME)**	**Structure**	**Fatty Acid Composition (wt**%** of total FAME)**	
		Soxhlet	SWE (Run 15)
Caprylic	C8:0	0.069	0
Capric	C10:0	0.062	0
Undecanoic	C11:0	0.848	0.737
Lauric	C12:0	0.334	0.990
Tridecanoic	C13:0	0.215	2.020
Myristic	C14:0	2.578	5.491
Pentadecanoic	C15:0	0.209	0.378
Cis-10-Pentadecenoic	C15:1	0	0.284
Palmitic	C16:0	18.031	29.269
Palmitoleic	C16:1	22.849	35.581
Margaric	C17:0	0.205	0.312
Cis-10-Heptadecenoic	C17:1	0.164	0
Stearic	C18:0	0.436	0.481
Oleic	C18:1n9c	3.566	3.386
Linoleic	C18:2n6c	2.315	1.914
*ɣ*-Linolenic	C18:3n6	0.337	1.072
*α*-Linolenic	C18:3n3	4.006	0
Cis-11,14-Eicosadienoic	C20:2	0.996	0
Cis-11,14,17-Eicosatrienoic	C20:3n3	0.763	1.037
Cis-8,11,14-Eicosatrienoic	C20:3n6	3.083	2.434
Arachidonic	C20:4n6	0.514	0
Eicosapentaenoic (EPA)	C20:5n3	28.103	14.135
Erucic	C22:1n9	6.393	0.478
Docosadienoic	C22:2	2.259	0
Tricosanoic	C23:0	1.664	0

**Saturated Fatty Acid (SFA) (wt**%** of total FAME)**		25.647	39.678
**Monounsaturated Fatty Acid (MUFA) (wt**%** of total FAME)**		32.973	39.730
**Polyunsaturated Fatty Acid (PUFA) (wt**%** of total FAME)**		42.376	20.592
**Omega-3 (wt**%** of total FAME)**		32.873	15.172
**Omega-6 (wt**%** of total FAME)**		6.249	5.420
**Lipid Yield (wt**%** of total biomass)**		10.200	17.860

**Table 5:** ANOVA of all factors and summary statistics of lipid yield.

Response variables	Source	Sum of squares	DF	Mean square	F-value	*p* value
Y_1_	Model	202.934	7	28.991	17.067	0.0002
	*Linear*					
	A	67.555	1	67.555	39.770	0.0001
	B	0.126	1	0.126	0.074	0.7915
	C	47.109	1	47.109	27.733	0.0005
	*Quadratic*					
	A^2^	42.431	1	42.431	24.979	0.0007
	B^2^	50.572	1	50.572	29.772	0.0004
	C^2^	17.097	1	17.097	10.065	0.0113
	*Interaction*					
	BC	15.015	1	15.015	8.840	0.0156
	Residual	15.288	9	1.699		
	Lack of Fit	12.770	7	1.824	1.449	0.4673
	Pure Error	2.517	2	1.259		
	Corrected Total	218.222	16			
	*Summary Statistics*					
	R^2^	0.9299				
	Adjusted R^2^	0.8755				
	Predicted R^2^	0.7381				
	Adequate Precision	12.1945				

A: temperature (°C), B: extraction time (min), and C: biomass loading (g algae/L)

**Table 6:** ANOVA of all factors and summary statistics of EPA composition.

Response variables	Source	Sum of squares	DF	Mean square	F-value	*p* value
*Y*_2_^−0.5^	Model	0.136	3	0.0429	42.389	< 0.0001
	*Linear*					
	A	0.084	1	0.084	78.358	< 0.0001
	C	0.005	1	0.005	4.703	0.0492
	*Quadratic*					
	A^2^	0.047	1	0.047	44.107	< 0.0001
	Residual	0.014	13	0.001		
	Lack of Fit	0.013	11	0.001	1.747	0.4199
	Pure Error	0.001	2	0.001		
	Corrected Total	0.150	16			
	*Summary Statistics*					
	R^2^	0.9073				
	Adjusted R^2^	0.8859				
	Predicted R^2^	0.8526				
	Adequate Precision	21.388				

A: temperature (°C), B: extraction time (min), and C: biomass loading (g algae/L)

**Table 7:** Optimization table for the specific condition and its desirability.

Condition	Temperature	Extraction	Biomass Loading	Lipid Yield	EPA Composition	Desirability
	(°C)	Time (min)	(g algae/L)	(wt% of biomass)	(wt% of total FAME)	
Maximize lipid yield (Predicted)	235.04	13.38	51.71	18.408	13.325	1.00
Maximize EPA composition (Predicted)	237.79	16.67	100.00	14.613	17.887	0.973
**Maximize lipid yield and EPA composition (Predicted)**	**236.54**	**13.95**	**60.50**	**18.278**	**14.036**	**0.934**
**Maximize lipid yield and EPA composition (Experimental)**	**236.54**	**13.95**	**60.50**	**13.405**	**15.040**	-

## Data Availability

All data is available within the article.
